# Effect of regular winter swimming on blood morphological, rheological, and biochemical indicators and activity of antioxidant enzymes in males

**DOI:** 10.1186/s13102-024-00932-3

**Published:** 2024-06-21

**Authors:** Aneta Teległów, Kamil Konieczny, Ignacy Dobija, Justyna Kuśmierczyk, Łukasz Tota, Konrad Rembiasz, Marcin Maciejczyk

**Affiliations:** 1 Department of Health Promotion, Institute of Basic Sciences, Faculty of Rehabilitation, University of Physical Education in Krakow, al. Jana Pawła II 78, Krakow, 31-571 Poland; 2 Graduate, Faculty of Rehabilitation, University of Physical Education in Krakow, al. Jana Pawła II 78, Krakow, 31-571 Poland; 3 Faculty of Physical Education and Sport, University of Physical Education in Krakow, al. Jana Pawła II 78, Krakow, 31-571 Poland; 4 Department of Physiology and Biochemistry, Faculty of Physical Education and Sport, University of Physical Education in Krakow, al. Jana Pawła II 78, Krakow, 31-571 Poland; 5 Indoor Swimming Pool Complex, University of Physical Education in Krakow, al. Jana Pawła II 78, Krakow, 31-571 Poland

**Keywords:** Antioxidant enzymes, Biochemical parameters, Cold water immersion, Haemorheology, Winter swimming

## Abstract

**Background:**

Exposure of the human body to cold water triggers numerous beneficial physiological changes. The study aimed to assess the impact of regular winter swimming on blood morphological, rheological, and biochemical indicators and activity of antioxidant enzymes in males.

**Methods:**

The study involved 10 male winter swimmers (the same participants examined before the season and after the season) and 13 males (not winter swimming, leading a sedentary lifestyle) in the control group. Fasting blood was collected twice: in November and in March of the following year. Basic blood morphological indicators, red cell elongation index (EI) and aggregation index (AI), concentrations of testosterone, cortisol, urea, and creatinine, as well as plasma activity of antioxidant enzymes of catalase (CAT), superoxide dismutase (SOD), and glutathione peroxidase (GPx) were determined.

**Results:**

The data were collected from the same winter swimmers at the beginning and end of the season. Winter swimming resulted in a significant increase of EI values at a shear stress of 0.30 (*p = 0.40*), 0.58 (*p < 0.001*), 4.24 (*p = 0.021*), 8.23 (*p = 0.001*), 15.59 (*p = 0.001*), 30.94 (*p = 0.004*), and 60.00 Pa (*p = 0.043*); haemoglobin was lower than before the season (*p < 0.027*). No significant changes were observed in AI, AMP, T1/2, the levels of urea, creatinine, eGFR, testosterone, cortisol, or the activity of CAT or SOD. There was a statistically significant increase in GPx activity (*p = 0.014*) and increase in testosterone concentration (*p = 0.035*) in the group of winter swimmers examined before the season as compared with the control group. No statistically significant differences were found for the mean values of blood morphological indicators and other parameters.

**Conclusions:**

Winter swimming can prove to be a health-promoting factor in males, as indicated by a rise in the deformability of red blood cells in the blood vessel system after a full season of winter swimming, leading to better body oxygenation, and improves the antioxidant defence and testosterone concentration (within standard limits) in the group of winter swimmers examined before the season as compared with the control group. Winter swimming helps maintain appropriate levels of blood rheological indicators, urea, creatinine, eGFR, cortisol, testosterone, and activity of antioxidant enzymes.

**Trial registration:**

ClinicalTrials.gov identifier NCT06223087, 15.01.2024.

## Introduction

Exposure of the human body to cold water triggers numerous physiological processes which constitute a defence mechanism to protect crucial human organs from hypothermia [1–6]. Scientific studies have confirmed that the bodies of people regularly subjected to cold water immersion activate adaptive processes in response to this stimulus, becoming more and more adapted over time [1, 7]. Winter swimmers exhibit metabolic, hypothermic, and insulative types of cold adaptation [8]. Greater resistance to stress and inflammatory responses have been reported in winter swimmers [9]. Cold water exposure creates an analgesic effect, does not induce addiction, and, most importantly, produces no side effects [10]. 

Beneficial impacts of winter swimming on both the mental (by reducing depression symptoms [11]) and physical spheres have been observed; these result from improvements in the haemorheological system, leading to a reduction in deviations from homeostasis [7, 12, 13]. Hormones have a significant impact on human behaviour and cognitive functions. Cold water awakens the brain with many electrical impulses. It increases the happiness hormones that instil a state of well-being and optimism in the individual. It reduces the level of cortisol, known as the stress hormone. It contributes to mental and physical relaxation [14, 15]. 

Since ancient times, mankind has known the advantageous effects of cold, which are applied in various areas of life. Nowadays, cold finds enormous popularity in physiotherapy and wellness [16, 17]. Its influence is widely exploited by winter swimmers, i.e. individuals who regularly bathe in cold waters in the period from late autumn to early spring. Being an extremely potent stimulus to the body, cold baths can provide a number of benefits, as well as risks if used inappropriately.

The presented study aimed to assess the impact of regular winter swimming on blood morphological, rheological, and biochemical indicators, as well as activity of antioxidant enzymes in males.

The following research hypothesis was put forward: winter swimming exerts a beneficial impact on selected blood morphological, rheological, and biochemical indicators, as well as activity of antioxidant enzymes in males.

## Materials and methods

### Characteristics of the study group

The study involved 10 male winter swimmers aged 43 ± 6.96 years from the Krakow Society of Winter Swimmers ‘Kaloryfer’ in Krakow (Poland) swimming from November to March, 3–5 min 1 time per week, for several years, each year a total of 22 times. The control group consisted of 13 males aged 44 ± 5.96 years who had never performed winter swimming and led a sedentary lifestyle.

Additionally, the participants were excluded due to the following: diabetes mellitus, the use of beta-blockers or anti-depressants, smoking or chewing tobacco products, and consuming more than 4 cups of coffee or more than 2 alcoholic beverages a day. The respondents were residents of Krakow (Poland), performing both physical and mental work. The subjects were included in the research programme after obtaining doctor’s consent and after physiotherapist consultation. Prior to enrolment in the study, each volunteer read the patient information leaflet; in case of doubt, they could ask questions, after which they gave their written informed consent to participate.

The majority of the participating winter swimmers reported attending weekly club meetings, during which they immersed themselves in water up to chest height, with their arms extending above the surface. The winter swimmers wore caps, gloves, and shoes while winter swimming. They communicated that the water temperature during the bathing did not exceed 10 °C, approaching 0 °C during the winter period. The winter swimming was practised at the Bagry Lagoon in Krakow.

On the day when fasting blood was collected from the winter swimmers, the air temperature was 5.3 °C, with water temperature of 6 °C. Blood samples were taken in the morning in the Blood Physiology Laboratory of the Central Research and Development Laboratory, University of Physical Education in Krakow, in November. During the second blood collection from the same winter swimmers, the air temperature was 4.5 °C, with water temperature of 4 °C. Fasting blood samples were also taken in the morning, in the same laboratory. This test was performed in March of the following year. Biochemical blood testing was conducted in the Diagnostyka laboratory in Krakow; in the Blood Physiology Laboratory of the Central Research and Development Laboratory, University of Physical Education in Krakow; and in the Genetic Analyses Laboratory of the Central Research and Development Laboratory, University of Physical Education in Krakow.

In the course of the research, 2 study groups were analysed:

- group 1: the control group;
- group 2: the group who practised winter swimming, examined before and after the season.

### Blood analysis methods

The blood samples were collected by a qualified nurse, from an ulnar vein, into EDTA K2 BD Vacutainer tubes and into Vacuette tubes with Serum Clot Activator.

Basic blood morphological indicators were measured with a Horiba ABX Micros 60 analyser.

Regarding blood rheological indicators, results concerning red blood cell aggregation and deformability were obtained by using the Laser-Assisted Optical Rotational Red Cell Analyzer (Lorrca) MaxSis, employing laser and optical technology. The data were analysed with the Hardeman method and the results were presented by means of the red cell elongation index (EI) and aggregation index (AI) [18]. 

Blood biochemical indicators were measured in blood serum with the use of Roche biochemical analysers. Testosterone concentration [ng/dl] was determined with a Cobas e 801 analyser. The Elecsys Testosterone II assay from Roche and the electrochemiluminescence immunoassay (ECLIA) method were applied. Cortisol concentration [µg/dl] (reference range in the morning hours of 6–10: 4.82–19.5 µg/dl) was detected with a Cobas e 602 analyser. The Elecsys Cortisol II assay from Roche and the ECLIA method were employed. Urea concentration [mmol/l] was established with a Cobas e 702 analyser. The kinetic method with urease and glutamate dehydrogenase was utilized. Creatinine concentration [µmol/l] was assessed with a Cobas e 702 analyser. Jaffe’s kinetic colorimetric method with picric acid was used.

Plasma activity of antioxidant enzymes was determined with the use of Cayman reagent kits: 707,002 for catalase (CAT), 706,002 for superoxide dismutase (SOD), and 703,102 for glutathione peroxidase (GPx).

### Statistical analysis

Data are presented as means and standard deviations (or, in the case of distributions that significantly deviated from normal, as medians and quartile deviations). The Shapiro-Wilk test was used to evaluate the distribution of variables. Equality of variance was checked using Levene’s test. The difference between the winter swimming group and the control group was assessed by using either Student’s t test or its non-parametric alternative, the Mann-Whitney U test. The difference in pre-post measurements of the winter swimming group was tested with either Student’s t test or its non-parametric alternative, the Wilcoxon paired rank order test. All tests were conducted with the standard significance level of α = 0.05. The analyses were carried out by using the Statistica 13 software package (StatSoft®, USA).

## Results

In the 2 analysed groups (the control group, the winter swimmers examined before the season and after the season), no statistically significant changes were found for the mean values of blood morphological indicators (Table 1). Only after winter swimming, haemoglobin was lower than before the season (*p < 0.027*).

**Table 1:** Morphological indicators of blood of control group and pre-post winter swimmers groups

parameters	control			winter swimmers pre			*p*	winter swimmers post			*p*
	mean	±	SD	mean	±	SD		mean	±	SD	
Leukocytes [10^9^/l]	5.88	±	1.01	6.33	±	1.96	0.480	5.29	±	1.27	0.079
Erythrocytes [mln/µl]	5.19	±	0.34	5.13	±	0.36	0.695	5.07	±	0.42	0.286
Haemoglobin [g/dl]	15.65	±	0.83	15.28	±	0.95	0.326	14.99	±	0.90*	0,027
Haematocrit [%]	45.30	±	2.33	44.26	±	2.71	0.334	43.46	±	2.88	0,0.76
MCV[fl]	87.47	±	2.77	86.41	±	3.56	0.430	85.85	±	3.49	0.339
MCH[pg]	30.22	±	0.98	29.82	±	0.79	0.310	29.63	±	0.96	0.250
MCHC[g/dl	34,56	±	0.84	34.53	±	0.86	0.930	34.53	±	1.12	1.000
Platelets [10^9^/l]	234.77	±	49.04	249.60	±	59.70	0.520	234.60	±	51.61	0.088
RDW-SD[fl]	39.49	±	1.66	38.98	±	2.48	0.559	38.46	±	1.44	0.449
RDW-CV[%]	12.46	±	0.43	12.44	±	0.36	0.899	12.54	±	0.33	0.372
Neutrophils [thousands/µl]	3.20	±	0.69	3.60	±	1.49	0.401	2.59	±	0.64	0.056
Lymphocytes [thousands/µl	2.01	±	0.37	1.91	±	0.64	0.661	1.93	±	0.61	0.891
Monocytes [thousands/µl]	0.49	±	0.10	0.57	±	0.12	0.110	0.49	±	0.14	0.136
Eosinophils [thousands/µl]	0.15	±	0.10	0.20	±	0.11	0.242	0.16	±	0.09	0.069
Basophils [thousands/µl	0.04	±	0.02	0.05	±	0.02	0.121	0.04	±	0.02	0.138

Abbreviations: MCV = mean corpuscular volume; MCH = mean corpuscular haemoglobin; MCHC = mean corpuscular haemoglobin concentration; RDW-SD = red blood cell distribution width standard deviation; RDW-CV = red blood cell distribution width coefficient of variation

Note: * difference compared to the measurement of swimmers before winter, statistically significant *p* < 0.05.

**Fig. 1:**
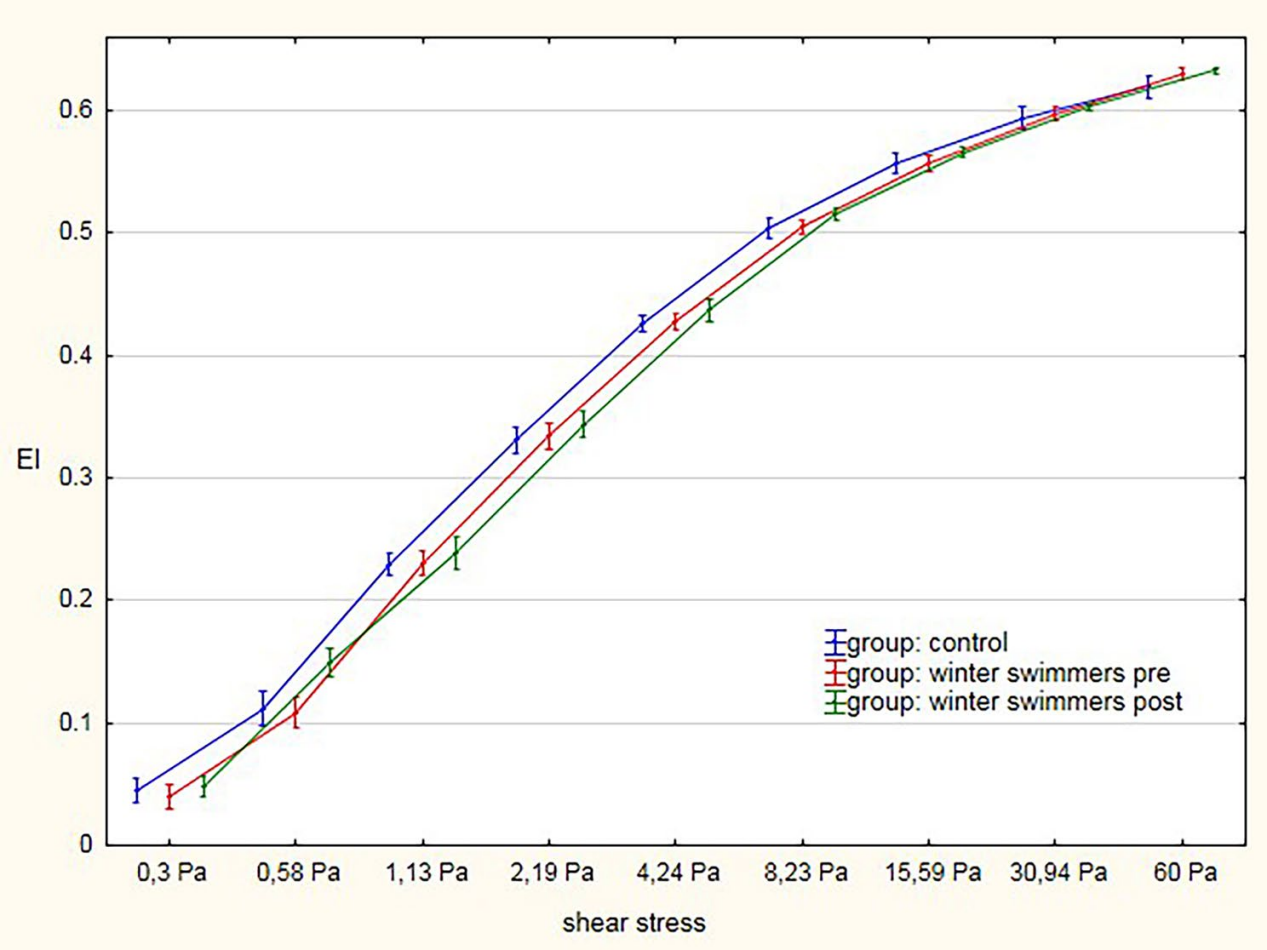
Elongation index (EI)–shear stress (SS) curves for RBC in studied groups. *p  <  0.05 compared tobaseline andcontrols

As for blood rheological indicators, EI displayed statistically significant increases after winter swimming at shear stress values of 0.30 (*p = 0.040*), 0.58 (*p < 0.001*), 4.24 (*p = 0.021*), 8.23 (*p = 0.001*), 15.59 (*p = 0.001*), 30.94 (*p = 0.004*), and 60.00 Pa (*p = 0.043*) (Table 1; Fig. 1) as compared with the winter swimmers before the season. There were no significant EI changes at a shear stress of 1.13–2.19 Pa.

There were no statistically significant changes in the blood rheological indicator EI in the group of winter swimmers examined before the season as compared with the control group. Only significant changes were observed for EI at shear stress of 60.00 (*p = 0.006*) (Fig. 1).

No statistically significant changes were reported for AI, erythrocyte aggregation amplitude (AMP), or half-time of total aggregation (T1/2) (Table 2).

**Table 2:** Blood rheological indicators of blood of control group and pre-post winter swimmers groups

parameters	control			winter swimmers pre			*p*	winter swimmers post			*p*
	mean	±	SD	mean	±	SD		mean	±	SD	
AMP [au]	37.10	±	2.72	38.89	±	3.29	0.168	38.00	±	2.80	0.585
AI [%]	61.77	±	8.75	58.66	±	8.78	0.409	57.93	±	6.40	0.756
T1/2 [s]	2.42	±	0.93	2.91	±	1.08	0.256	2.95	±	0.80	0.906
EI at 0.3 Pa	0.045	±	0.010	0.039	±	0.010	0.196	0.048	±	0.008 *	0.040
EI 0.58 at Pa	0.112	±	0.015	0.109	±	0.012	0.591	0.149	±	0.012 ***	< 0.001
EI 1.13 at Pa	0.230	±	0.010	0.231	±	0.010	0.794	0.238	±	0.013	0.155
EI 2.19 at Pa	0.331	±	0.011	0.335	±	0.011	0.396	0.344	±	0.011	0.058
EI 4.24 at Pa	0.426	±	0.007	0.428	±	0.007	0.527	0.438	±	0.009 *	0.021
EI 8.23 at Pa	0.504	±	0.009	0.505	±	0.005	0.708	0.515	±	0.005 **	0.001
EI 15.59 at Pa	0.558	±	0.009	0.558	±	0.007	0.986	0.566	±	0.005 **	0.001
EI 30.94 at Pa	0.594	±	0.009	0.597	±	0.006	0.314	0.603	±	0.003 **	0.004
EI 60 Pa at Pa	0.620	±	0.009	0.630	±	0.005 ##	0.006	0.633	±	0.002 *	0.043

Note:

# difference from the control group statistically significant *p* < 0.05

# # difference from the control group statistically significant *p* < 0.01

* the difference from the measurement before was statistically significant *p* < 0.05

** the difference from the measurement before was statistically significant *p* < 0.01

*** the difference from the measurement before was statistically significant *p* < 0.001

Abbreviations: AMP = erythrocyte aggregation amplitude; T1/2 = half-time of total aggregation; AI = aggregation index; EI = elongation index

Statistically significant increases in testosterone concentration [ng/dl] (*p = 0.035*) were observed in the group of winter swimmers examined before the season as compared with the control group (Table 3).

**Table 3:** Testosterone concentration of blood of control group and pre-post winter swimmers groups

parameter	control			winter swimmers pre			*p*	winter swimmers post			*p*
	mean	±	SD	mean	±	SD		mean	±	SD	
Testosteron	407.00	±	115.30	651.50	±	304.68	0.035#	652.60	±	272.86	0.986

Note:

# difference from the control group statistically significant *p* < 0.05

With regard to the blood biochemical indicators of urea [mmol/l], creatinine [µmol/l], cortisol [µg/dl], and eGRF concentrations, no statistically significant changes were revealed between the control group and the group of winter swimmers examined before or after the season (Table 4).

**Table 4:** Blood biochemical indicators of control group and pre-post winter swimmers groups

Parameters	control			winter swimmers pre			*p*	winter swimmers post			*p*
	mean	±	SD	mean	±	SD		mean	±	SD	
Urea[mmol/l]	5.18	±	1.18	5.80	±	2.03	0.365	5.75	±	2.02	0.896
Creatinine [µmol/l]	89.08	±	12.67	83.70	±	6.77	0.239	80.10	±	8.29	0.095
Cortisol [µg/dl]	12.87	±	3.58	13.65	±	2.28	0.552	15.00	±	2.85	0.390
eGRF	81.11	±	11.24	86.06	±	5.36	0.180	88.46	±	4.60	0.232

No statistically significant changes were reported in the activity of CAT or SOD. However, there were differences in the activity of GPx (*p = 0.029*). The activity of GPx statistically significantly increased in the group of winter swimmers examined before the season as compared with the control group (*p = 0.035*) (Table 5).

**Table 5:** Activity of catalase, superoxide dismutase and glutathione peroxidase (mean values) of blood of control group and pre-post winter swimmers groups

parameters	control			winter swimmers pre			*p*	winter swimmers post			*p*
	mean	±	SD	mean	±	SD		mean	±	SD	
GPx [nmol/min/ml]	81.17	±	26.40	107.72	±	18.18 #	0.014	97.92	±	20.66	0.306
SOD [U/ml]	1.18	±	0.31	0.89	±	0.45	0.084	1.03	±	0.50	0.333
CAT [nmol/min/ml]	8.50	±	4.50	10.94	±	3.44	0.177	9.64	±	5.21	0.565

# difference from the control group statistically significant *p* < 0.05

Abbreviations: CAT = catalase; SOD = superoxide dismutase; GPx = glutathione peroxidase

## Discussion

Winter swimming is among the components that affect the rheological properties of blood [7, 19]. The study was conducted to determine the magnitude of the impact of the cold factor on blood morphological, rheological, and biochemical properties, as well as the activity of antioxidant enzymes in males. The potential of erythrocytes to modify their structure is crucial during their passage through the tiny capillaries. Red cell deformation is associated with essential changes in the protein structure of the cytoskeleton, the physical properties of the lipid membrane, and haemoglobin concentration [20]. Rheological blood testing measures 4 basic erythrocyte characteristics: deformability (EI), AI, plasma viscosity, and blood viscosity. Erythrocytes exploit their deformability potential to fulfil their function and squeeze through capillaries. The modifications do not affect the volume or surface area of the blood cell itself; therefore, when passing into a larger diameter vessel, erythrocytes return to their previous shape. A key determinant of cell wall stiffness in red cells is haemoglobin, which conditions the normal cell shape and the intracellular viscosity. Thus, haemoglobin concentration plays an extremely important role in the ability of the erythrocyte to deform [21, 22]. 

Regular winter swimming of men aged 43 ± 6.96 years for a period of 4 months produced a positive effect on blood rheological properties, which points to an efficient haemorheological system interacting with the cardiovascular system. In the study, a statistically significant increase in EI values was observed at the shear stress values of 0.30, 0.58, 4.24, 8.23, 15.59, 30.94, and 60.00 Pa after the entire winter swimming season. These findings corroborate a study by Teległów et al. [7]. , which revealed a positive effect of winter swimming on blood rheological properties, manifested by an increase in erythrocyte deformability without accompanying changes in erythrocyte aggregation. Increased deformability of red blood cells in a constricted vascular system is a safeguard to facilitate erythrocyte flow. For morphological indicators, the study did not show statistically significant changes; only after the winter swimming, haemoglobin was lower than before the season. Aggregation, in turn, consists in the formation of aggregates (rouleaux formation). It occurs naturally and is susceptible to shear forces. The factors influencing the formation of aggregates can be divided into 2 groups: external and internal. The former involve plasma protein concentrations, haematocrit level, and shear forces. The latter include red blood cell shape and deformability, as well as the properties of lipid membrane surface. Another crucial factor that accelerates aggregation is fibrinogen. A decrease in its plasma concentration, like a rise in erythrocyte count, leads to an increase in haematocrit concentration and, consequently, to a higher viscosity [23]. In the present study, no statistically significant changes were observed in the values of red blood cell aggregation indicators, i.e. AMP, T1/2, and AI. This can be explained by an efficient haemorheological system interacting with the cardiovascular system [23]. These results confirm those obtained by Teległów et al. [7]. 

The study demonstrated that winter swimming did not increase testosterone levels after the season. This is most likely because the body accustoms to low temperature. There was a statistically significant increase in testosterone concentration in the group of winter swimmers examined before the season as compared with the control group. This indicates the struggle of the body to survive in exceptionally difficult conditions [24]. As reported by Teległów et al. [1]. , a 50-day exposure to extremely low air temperatures (from − 5 °C to − 37 °C in Rovaniemi, a city in northern Finland) resulted in a 60.14% increase in testosterone concentrations in the examined subject. The study participant spent time being active, walking at a distance of about 20 km every day (mountain walking), skiing, and cycling on ice to avoid freezing; this also increases testosterone levels, as previously discussed by Fujibayashi and Yoshida [25], as well as by Ismail and Young [26]. A long-term process of winter swimming should be furthermore perceived in terms of physical effort; each time the body cools down, its homeostasis is disrupted, and additional energy is required to compensate for this. It benefits specific thermoregulatory processes, the circulatory system, and the immune system. In addition, it lowers the concentration of cholesterol, from which testosterone is synthesized, which, apart from the harsh conditions induced by several months of cooling the body in cold water, can also result in an increase in testosterone levels. A more detailed look at the characteristics of these changes may shed light on new approaches to address low testosterone levels in males, which can have devastating effects on their lives by contributing to insomnia, increased body fat, decreased muscle mass, sexual dysfunction, and depression [24, 27]. The present study revealed no statistically significant changes for urea, creatinine, eGFR, testosterone, or cortisol levels either after a full season of winter swimming or in comparison with the control group. No changes in these indicators were also reported by Teległów et al. [1, 2, 7]. , Selleri et al. [28]. , Knechtle et al. [29]. , and Drygas et al. [30]. , which confirms that repeated exposure to low temperatures enhances the adaptation of the body to the prevailing conditions. In turn, Hermanussen et al. [31] observed a rise in cortisol concentration 30 min after leaving the water and a significant decrease 60 min after leaving the water.

It is known that low temperature stress leads to increased oxygen consumption, which results in oxidative stress, harmful to red blood cells. Erythrocytes, like other cells, are equipped with a strong antioxidant defence in the form of the SOD, GPx, and CAT enzymes. Oxidative stress can decrease erythrocyte deformability and increase erythrocyte aggregation, contributing to reduced efficiency in oxygen delivery to tissues. However, Siems et al. [32] reported no changes in the activity of CAT, SOD, or GPx in the erythrocytes of regularly winter swimming individuals. The lack of statistically significant changes for CAT and SOD activity observed in the present study after the entire season of winter swimming confirms that regular cold exposure improves the antioxidant defence of the body in males.

### Limitation of the study

In our study, the control group was tested only once, at the beginning of the follow-up. An assumption was made that in healthy people (such as our participants), the values of the indicators studied would not show significant changes if they were not subjected to any intervention.

## Conclusions

Winter swimming can prove to be a health-promoting factor in males, as indicated by a rise in the deformability of red blood cells in the blood vessel system after a full season of winter swimming, leading to better body oxygenation, and improves the antioxidant defence and testosterone concentration (within standard limits) in the group of winter swimmers examined before the season as compared with the control group.

## Data Availability

No datasets were generated or analysed during the current study.
